# Application of DNA-based diagnostics in detection of schistosomal DNA in early infection and after drug treatment

**DOI:** 10.1186/1756-3305-4-164

**Published:** 2011-08-24

**Authors:** Cen Wang, Lin Chen, Xuren Yin, Wanquan Hua, Min Hou, Minjun Ji, Chuanxin Yu, Guanling Wu

**Affiliations:** 1 Department of Pathogen Biology & Immunology, Nanjing Medical University, Nanjing 210029, China; 2 Jiangsu Institute of Parasitic Diseases, Wuxi 214064, Jiangsu, China

## Abstract

**Background:**

Research is now focused on identification of sensitive and specific diagnostic tests for early identification of schistosomal infection and evaluation of chemotherapy in field situations in China.

**Results:**

This study compared loop-mediated isothermal amplification (LAMP) with conventional PCR as DNA-based diagnostic techniques for the early detection of schistosomal DNA and the evaluation of chemotherapy. The results showed that both PCR and LAMP assays targeting a 301 base pair (bp) sequence of the highly repetitive retrotransposon, SjR2, amplified DNA from schistosomes but were unable to distinguish between schistosome species. LAMP and conventional PCR were shown to amplify the target sequence of the SjR2-pCR2.1 recombinant plasmid template with limits of detection of 10^-4 ^ng and 10^-2 ^ng, respectively, thus demonstrating the superior sensitivity of the LAMP method. *Schistosoma japonicum *DNA was detected in all serum samples obtained from the three experimental groups at 1 week post-infection by LAMP assay, while the rate of detection by conventional PCR ranged from 50% to 66%. The potential application of PCR and LAMP assays for the evaluation of artesunate and praziquantel chemotherapy was investigated. PCR was shown to be less sensitive for detection of schistosomal DNA in drug-treated rabbit sera than the LAMP method.

**Conclusions:**

The data presented here indicate that LAMP is suitable for the detection of early infection in the groups primarily infected with *Schistosoma japonicum*, such as migrants, travellers, military personnel and the younger age groups. However, it is less suitable for evaluation of the efficacy of chemotherapy in the early stages because of its high sensitivity.

## Background

Schistosomiasis remains one of the most common parasitic diseases, afflicting more than 200 million people worldwide [1]. In China, *Schistosoma japonicum *is the only causative species of schistosomiasis, which leads to hepatic periportal fibrosis and portal hypertension due to the deposition of *Schistosoma japonicum *eggs in tissues[2]. The morbidity associated with schistosomiasis has been successfully controlled in China through chemotherapy. However, it is difficult to eliminate this disease completely in endemic areas and the epidemiologic situation persists at a low level both in prevalence and the intensity of infection. Furthermore, schistosomiasis is an emerging problem in non-endemic areas due to broader distribution of snails and increased immigration and tourism etc. [3,4] In order to address this issue, research is now focused on identification of sensitive and specific diagnostic tests for early identification with *Schistosoma japonicum *and evaluation of chemotherapy in field situations in China.

The Kato-Katz method is the currently used 'gold standard' technique against which novel diagnostic tests are evaluated. However, this method relies on the detection of eggs in stool samples which are not released into the intestinal lumen until 25 to 26 days after infection with *Schistosoma japonicum*[5]. Consequently, this direct parasitological detection technique is associated with poor sensitivity which limits both the diagnosis of individuals with early or low level infections and its application in evaluation of the efficacy of chemotherapy. Immunodiagnostic techniques which are more sensitive and simpler to perform have become a common epidemiological tool for screening target populations in many schistosome-endemic areas. However, detection antibodies lack specificity[6-8] and immunodiagnostic techniques such as circumoval precipitin test (COPT), indirect haemagglutination test (IHA) and enzyme linked immunosorbent assay (ELISA) used for chemotherapy evaluation, are associated with high positive rates for detection of schistosomal antibodies for a long time (40.2%-41.2%, 1 to 2 years post-treatment and 4.26% - 17.5% after at least 3 years of treatment) [9]. This limits the application of immunodiagnostics for detection of infection and evaluation of chemotherapy.

It has been reported that assays based on polymerase chain reaction (PCR) techniques are capable of detecting DNA released from *Schistosoma mansoni, Schistosoma haematobium *and *Schistosoma japonicum *[10-14]. Xia et al. described a PCR assay for amplification of a 230-bp sequence from the highly repetitive retrotransposon, SjR2, of *Schistosoma japonicum *in rabbit serum one week after infection. This test turned negative at 10 weeks post-treatment with, praziquantel although levels of schistosome-specific IgG remained at a high level up to 23 weeks post-treatment [15].

More recently, LAMP, a simple, rapid and sensitive detection technique has been established [16]. This method uses *Bst *DNA polymerase (Highest strand displacement activity available, New England Biolabs) with strand displacement activity for amplification in less than one hour under isothermal conditions. This technique has been used to detect pathogens, such as African trypanosomes [17], *Babesia orientalis *[18], *Cryptosporidium*[19], *Plasmodium*[20,21], *Toxoplasma gondii *[22] and schistosomes [23,24].

In this study, the sensitivity and specificity of conventional PCR and LAMP assays were compared for the detection of a 301-bp sequence derived from the sequence of SjR2 of *Schistosoma japonicum *genomic DNA in blood samples obtained from experimentally infected rabbits. Furthermore, the potential applications of these two techniques for early diagnosis and an evaluation of the efficacy of chemotherapy were explored.

## Results

### Sensitivity and specificity of LAMP and PCR assays

**Figure 1:**
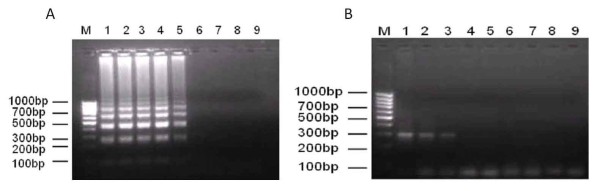
**Sensitivity of the LAMP and PCR methods for detection of recombinant plasmid of SjR2-pCR2.1**. (A) The LAMP assay method showed the higher sensitivity (10^-4 ^ng) than the PCR method. (B) PCR method was just able to detect more than 10^-2 ^ng of plasmid DNA. M: DNA marker; 1-8: 1 ng-10^-7^ng plasmid DNA; 9: negative control without plasmid DNA.

**Figure 2:**
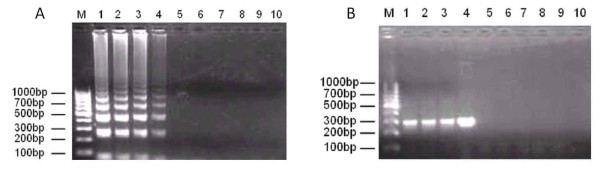
**Specificity of the LAMP and PCR methods using genomic DNA from different species samples**. (A) Specificity of LAMP method. (B) Specificity of PCR method. 1-10: The recombinant plasmid of SjR2-pCR2.1, *Schistosoma mansoni, Schistosoma japonicum *(Philippine strain), *Schistosoma japonicum *(Chinese mainland strain), *Clonorchis sinensis, Oncomelania hupensis, Plasmodium vivax, Plasmodium falciparum*, uninfected rabbits and negative controls.

A limit of detection of 10^-2 ^ng DNA was achieved by conventional PCR while higher sensitivity was achieved by LAMP assay with a lower limit of detection of 10^-4 ^ng DNA when the SjR2-pCR2.1 recombinant plasmid was used as template (Figure 1). Furthermore, the target sequence was successfully amplified from genomic DNA derived from *Schistosoma japonicum *(Chinese mainland strain), *Schistosoma japonicum *(Philippine strain), and *Schistosoma mansoni*. However, amplification of the sequence was not detected in *Clonorchis sinensis, Plasmodium vivax, Plasmodium falciparum, Oncomelania hupensis *or uninfected animals (normal rabbit whole blood sample) (Figure 2).

### Detection of early Schistosoma japonicum infection by LAMP and PCR assays

**Figure 3:**
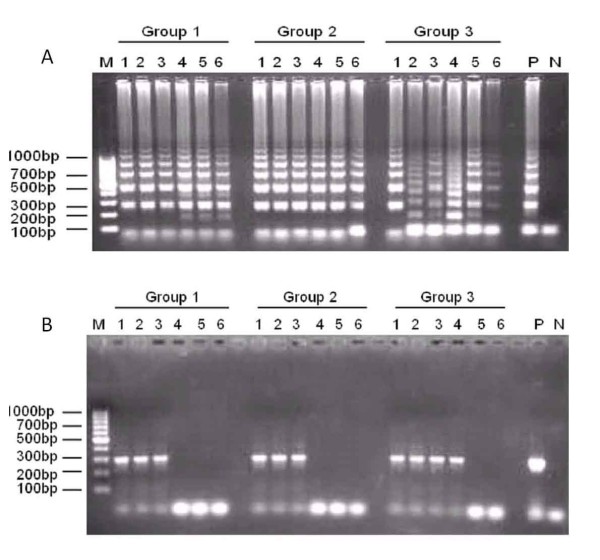
**Detection of early *Schistosoma japonicum *infection in rabbits by LAMP and PCR asay**. (A) The LAMP assay detected all the blood samples at 1 week after *Schistosoma japonicum *infection. (B) PCR method just detected partial blood samples at 1 week post-infection.

Detection of schistosomal DNA in early *Schistosoma japonicum *infection was investigated by conventional PCR and LAMP assays. Genomic DNA from rabbits before, and one week after *Schistosoma japonicum *infection was amplified using both methods. The LAMP assay detected schistosomal DNA in all blood samples from infected rabbits (200 or 1,500 cercariae) one week after infection (Figure 3A), indicating that the sufficient DNA was obtained for detection at the stage of early infection using the LAMP method. In contrast, detection by conventional PCR was less sensitive. Only 50% of blood samples were tested positive in Group 1 and Group 2, and 66% in Group 3 one week after infection using this method (Figure 3). All blood samples obtained two weeks post-infection in 3 groups tested positive by conventional PCR (Figure 5). Genomic DNA samples from uninfected rabbits were confirmed to be negative for *Schistosoma japonicum *DNA using both methods.

### Evaluation of the efficacy of drug treatment against schistosomiasis in rabbits

**Figure 4:**
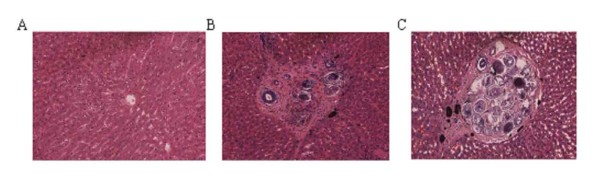
**Liver pathology with H&E staining in rabbits after artesunate or praziquantel treatment**. (A) Representative image of the liver section of Group 1 (× 200); (B and C) representative image (× 200) of egg granuloma in the liver section of Group 2 and Group 3, respectively.

Group 1 was treated orally with artesunate at 1 week post-infection. Group 2 and Group 3 were treated orally with praziquantel at 5 weeks post-infection. All rabbits were sacrificed at 21 weeks post-infection for investigation of the parasitological parameters. All rabbits were shown to be free from adult worms (Table 1), indicating that artesunate and praziquantel had effectively eliminated the schistosomula and adult worms respectively. The left lobe of the liver from each rabbit was analyzed histologically. Many typical egg granulomas were observed in the liver sections of Group 2 and Group 3 while none were observed in the liver sections of Group 1 (Figure 4). Furthermore, a greater number of eggs and egg granulomas were observed in Group 3 compared with Group 2.

**Table 1:** The parasitological parameters in rabbits following artesunate or praziquantel treatment

	Number of rabbits	Worms recovered	Number of egg in the liver section	Number of egg granulomas in the liver section
Group 1	6	-	-	-
Group 2	6	-	185	82
Group 3	6	-	966	109

**Figure 5:**
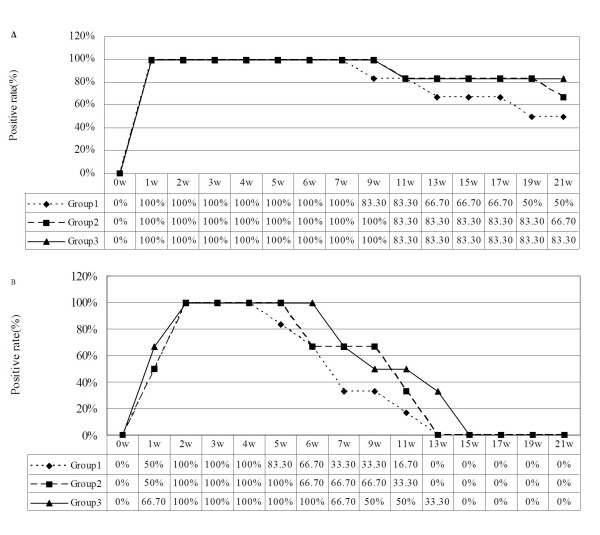
**Dynamic detection of schistosomal DNA in infected rabbits following artesunate or praziquantel treatment**. (A) Dynamic results by LAMP method. (B) Dynamic results by PCR method.

### Dynamic detection by LAMP and PCR assays of schistosomal DNA in infected rabbits following artesunate or praziquantel treatment

Despite the absence of eggs and adult worms observed in Group 1, schistosomal DNA was persistently detected in 100% of blood samples obtained 7 weeks post-infection (6 weeks after artesunate therapy) by the LAMP method (Figure 5A). The rate of detection was reduced to 50% at 21 weeks post-infection (20 weeks after artesunate therapy). Adult worms were eliminated in Group 2 and Group 3 by praziquantel treatment although eggs persisted for extended periods. Schistosomal DNA was detected in 100% of blood samples obtained from both groups (2 and 3) up to 9 weeks post-infection (4 weeks after praziquantel therapy), and 83% of blood samples up to 19 weeks post-infection (14 weeks after praziquantel therapy). Schistosomal DNA was detected in 66% of blood samples from Group 2, and 83% of blood samples in Group 3, as late as 21 weeks post-infection (16 weeks after praziquantel therapy).

In comparison with the LAMP method, positive detection of schistosomal DNA by conventional PCR was considerably less sensitive (Figure 5B). A detection rate of 100% in Group 1 was maintained up to 4 weeks post-infection only (3 weeks after artesunate therapy). This rate then declined rapidly until detection was no longer positive at 13 weeks post-infection (12 weeks after artesunate therapy). A similar pattern of detection was observed in Group 2 and Group 3. Schistosomal DNA was detected in all blood samples from 2 weeks to 5 weeks post-infection in Group 2, and up to 6 weeks post-infection in Group 3. All blood samples tested negative at 13 weeks post-infection (8 weeks after praziquantel therapy) in Group 2 and 15 weeks post-infection (10 weeks after praziquantel therapy) in Group 3, following analysis by conventional PCR.

At the same time, we also performed LAMP and PCR on positive (ongoing infection) and negative (uninfected) controls. Our results showed that detection of *Schistosoma japonicum *DNA in rabbits infected with 200 or 1500 cercariae for 4 and 21 weeks by these two methods was positive, while both LAMP and PCR did not detect *Schistosoma japonicum *DNA in all the samples of uninfected rabbits (negative control), data not shown here.

## Discussion

*Schistosoma japonicum *infection is well-controlled in China although eradication has not been achieved and re-emergence remains a threat. The availability of an effective diagnostic test is critical for detection of infection in the early stages and for evaluation of therapies. Effective primer design and gene targeting is crucial for specific DNA amplification by conventional PCR and LAMP methods. The repetitive nature of non-LTR retrotransposons, such as SjR2, and 28S ribosomal DNA components of the schistosome genome provides a suitable basis for targeting of these genes [19,25,26] In this study, a 301-bp sequence of SjR2 was targeted for comparison of the sensitivity of conventional PCR and LAMP assays. Results showed that the target sequence of the SjR2-pCR2.1 recombinant plasmid template was detected by LAMP at a lower limit of 10^-4 ^ng. However, conventional PCR was unable to detect the plasmid at concentrations below 10^-2 ^ng. These results are in accordance with previous reports [16,19,27-29]. However, the primers used in this study were not suitable for the purposes of distinguishing between *Schistosoma mansoni *and *Schistosoma japonicum*. Xu et al. reported amplification of a 230-bp sequence of SjR2 from adult worm and fecal egg DNA possessing approximately 10^4 ^copies of this sequence. The minimum level of detection of *Schistosoma japonicum *DNA by PCR assay was 0.8 pg and 0.08 fg by LAMP assay [26,28]. However, results of experiments targeting 28S ribosomal DNA have shown that conventional PCR and LAMP assays detected genomic DNA with the same sensitivity (100 fg) and that DNA from *Schistosoma japonicum *but not *Schistosoma mansoni *is amplified by both methods [25].

Following infection with *Schistosoma japonicum*, nucleic acids in the serum are derived mainly from epidermal tissue of migrating schistosomula and worm decomposition products[30,31]. The results of this study showed LAMP to be a more sensitive assay than conventional PCR for early diagnostic purposes. *Schistosoma japonicum *DNA was detected by LAMP assay in all serum samples of all three experimental groups at 1 week post-infection. However, a lower rate of detection (50%-66%) was observed by conventional PCR. At present, schistosomiasis was diagnosed based on the detection of parasite eggs in fecal samples up to weeks four to five post-infection, and by immunological methods based on detection of anti-schistosome antibodies, up to at least 2 weeks post-infection [15]. Therefore, LAMP assay for the detection of schistosome-derived DNA inverted in blood sample is the method of choice for early diagnosis of infection.

The detection of anti-schistosomal antibodies is of limited value for the diagnosis of infection and evaluation of chemotherapy at early time points due to antibody persistence and serological cross-reactivity [32-34]. Xia et al. reported detection of high levels of serum anti-worm IgG by ELISA at 23 weeks post-treatment with praziquantel for elimination of the *Schistosoma japonicum *adult worm in rabbits [15]. In this study, conventional PCR and LAMP assays were compared for their potential in early evaluation of chemotherapy regimens. Three groups of rabbits were separately administered artesunate or praziquantel to eliminate schistosomula and adult worms. All serum samples were tested by LAMP and PCR assay prior to infection and every successive week or every two weeks after infection. Schistosomal DNA in rabbit sera became undetectable by conventional PCR at 12 weeks post-therapy with artesunate in Group 1, at 8 weeks post-therapy with praziquantel in Group 2 and at 10 weeks post-therapy with praziquantel in Group 3. These results were in accordance with those reported by Xia et al., in which *Schistosoma japonicum *DNA in sera became undetectable by PCR at 10 weeks post-treatment with praziquantel [15]. Interestingly, *Schistosoma japonicum *DNA remained detectable in 50% of blood samples up to 20 weeks post-therapy with artesunate in Group 1, 66% in Group 2 and 83% in Group 3 at 14 weeks post-therapy with praziquantel by LAMP assay. The reason is although schistosomula or adult worms were eliminated by artesunate or praziquantel, nucleic acids in serum can be derived from egg decomposition products[35,36]. *Schistosoma japonicum *DNA persisted in serum for extended periods. The results of this study demonstrated the superior sensitivity of the LAMP assay compared with conventional PCR for evaluation of the efficacy of chemotherapy, which leads to the positive results for a longer duration with LAMP assay than PCR assay after chemical treatment. Thus, a positive detection by LAMP assay for an individual from an endemic area indicated there has been an infection but does not distinguish the infection status and the efficacy of chemical treatment. A shortcoming of the study is that all animals were sacrificed at 21 weeks post-infection, the experiments should have been carried on until schistosomal DNA was undetectable in all samples, thus we can tell the drugs indeed have worked according to the sensitivity of LAMP assay. This problem remains to be resolved.

We recognize that the aim of this study is to establish a new sensitive and specific diagnostic system for early identification of schistosome infection and evaluation of chemotherapy in field situations in China. It should be noted that amplification of nucleic acids by LAMP assay is carried out under isothermal conditions which therefore eliminates the requirement for more expensive thermal cycler equipment. LAMP assay has more obvious advantages in field research where experimental conditions are not ideal. However, our results show that the LAMP assay is suitable for diagnosis of those groups such as migrants, travelers, military personnel who participate in fighting a flood or the younger age groups in endemic areas who are primarily infected with *Schistosoma japonicum*, and likely the intensities of infection are at low level, which need to be diagnosed early by methods with high sensitivity and specificity. Furthermore, the evaluation of chemotherapy in field situations in China by LAMP assay still needs more in-depth research to assess it. Well-designed field studies in schistosomiasis endemic areas are desired which need to take into consideration epidemic season, status of infection, treatments received, and so on in the field.

## Conclusions

Targeting of the 301-bp sequence of SjR2 by LAMP assay is suitable for the detection of early infection in the groups primarily infected with *Schistosoma japonicum*, such as migrants, travellers, and the younger age groups in endemic areas. However, it has limitations for evaluation of the efficacy of chemotherapy in the early stages somewhat because of its high sensitivity. Targeting of this sequence by conventional PCR was shown to be less sensitive than LAMP for early detection of schistosomal DNA in blood samples, although it seems to be valuable for the evaluation of chemotherapy.

## Methods

### Sensitivity and specificity of LAMP and PCR assays

The SjR2-specific fragment derived from genomic DNA of *Schistosoma japonicum *(adult worm) was amplified by PCR using primers, F3 and B3(PCR conditions were described below). The resulting PCR product was then subcloned into the T-A vector, pCR2.1 (TA Cloning^® ^Kit with pCR^®^2.1 vector, Invitrogen) to generate the recombinant plasmid, which was used as the positive control. Amplification of the 301-bp insert in the SjR2-pCR2.1 recombinant plasmid template (Plasmid DNA was purified by QIAprep Spin Miniprep Kit. the amount of DNA was measured by spectrophotometer, PCR conditions as below)was carried out with a successive titration of template ranging from 1 ng to 10^-7 ^ng (10°ng, 10^-1 ^ng, 10^-2 ^ng, 10^-3 ^ng, 10^-4 ^ng, 10^-5 ^ng, 10^-6 ^ng, 10^-7 ^ng) in order to compare the sensitivities of the LAMP and conventional PCR methods.

The target 301-bp sequence was amplified from genomic DNA derived from *Schistosoma japonicum *(Chinese mainland strain), *Schistosoma japonicum *(Philippine strain), *Schistosoma mansoni, Clonorchis sinensis, Plasmodium vivax, Plasmodium falciparum *and *Oncomelania hupensi *to compare the specificity of the LAMP and conventional PCR methods, the PCR's conditions were the same as described below.

### Experimental infection with Schistosoma japonicum

Japanese White Rabbits were obtained from Jinling Animal Center (Nanjing, China) and housed at Jiangsu Institute of Parasitic Disease (Wuxi, China) under specific pathogen-free conditions. All experiments were performed in accordance with protocols approved by the Institutional Animal Care and Use Committee at Nanjing Medical University. Cercariae were collected from *Oncomelania hupensis *snails, which were laboratory-infected with the Chinese mainland strain of *Schistosoma japonicum*. Japanese White Rabbits (18) were divided into three groups (n = 6). Group 1 and Group 2 were percutaneously infected with 200 *Schistosoma japonicum *cercariae applied to the shaved abdomen. Group 3 were infected in the same way with 1,500 cercariae. At the same time we set up two positive control groups(ongoing infection without treatment):one group(n = 5) infected with 200 *Schistosoma japonicum *cercariae, the other group(n = 5) were infected with 1,500 cercariae, and negative control group(n = 2, uninfected and without treatment), data not shown.

### Treatment of infected rabbits and sample collection

**Figure 6:**
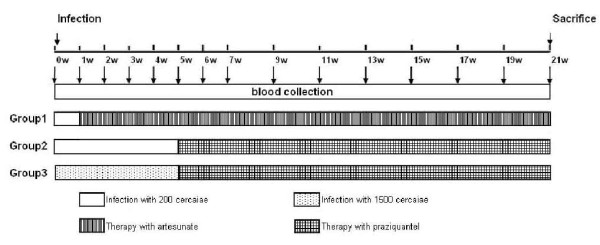
**Experiment schedule of infection, drug treatment and blood collection**.

Before infection, blood samples of all 18 rabbits were collected. After 1 week of the infection, Group 1 was administered 160 mg/kg/d artesunate (capsuled) orally for two consecutive days (8 and 9 days after infection). After 5 week of infection, Group 2 and Group 3 was administered 150 mg/kg/d praziquantel (capsuled) orally for two consecutive days (36 and 37 days after infection) (Figure 6). Blood samples (collected from the auricular vein, anticoagulant-treated, 1 ml) of all rabbits were collected as scheduled (Figure 6).

After 21 weeks of infection, all rabbits were sacrificed for investigation of the parasitological parameters. After perfusion of the thoracic aorta, the recovery of worms was determined by sedimentation of the perfusate plus residual worms from the intestinal mesenteric vessels. The left liver lobe was removed from all animals, fixed (10% buffered formalin) and embedded in paraffin. Sections (4 μm) were stained with H&E (hematoxylin and eosin) and examined for schistosome egg granulomas by light microscopy. The numbers of eggs and egg granulomas were calculated in three sections (approximately 10 mm × 5 mm) of a single liver from each animal (n = 18).

### DNA extraction

Total genomic DNA of whole blood collected from infected (Figure 6) and uninfected animals was extracted according to the protocol provided by the manufacturer (Qiagen QIAamp DNA Blood Midi Kit) and resuspended in 20 μl ddH_2_O.

*Schistosoma japonicum *(Chinese mainland strain), *Schistosoma japonicum *(Philippine strain), *Schistosoma mansoni, Clonorchis sinensis, Plasmodium vivax, Plasmodium falciparum *and *Oncomelania hupensi *genomic DNA were provided by Jiangsu Institute of Parasitic Disease (Wuxi, China)

### LAMP and PCR assays

The SjR2 sequence (GenBank Accession No. AY027869, 2719~3019 bp) of *Schistosoma japonicum *was selected as the target sequence for amplification by LAMP and PCR assays. The primers were designed by Primer Explore software as Table 2.

**Table 2:** Specific primers for the LAMP and PCR assay used in this study

**PCR**	F3: 5'- ACTTCTAGTGGTGTTCGTCAGGCTTGT-3'
	B3: 5'-CTAACTTTGGTGCCGAATTAAGCCA-3'
**LAMP**	F3: 5'-ACTTCTAGTGGTGTTCGTCAGGCTTGT-3'
	B3: 5'-CTAACTTTGGTGCCGAATTAAGCCA-3'
	FIP: 5'-AGGGAAATCAGACGATGACAATGCTATCTCCATTTTTATTTAA-3'
	BIP: 5'-TTTGACCACCTTAAACATGAATGAAGTAACATTTTACATTTGGA-3'
	LPF: 5'-TCTAAAAGTATGTCAATGATAA-3'
	LPB: 5'-AAGCATGCTTGGGATGCGATTCTC-3'

For LAMP assays, 12.5 μl of 2 × reaction buffer (40 mM Tris-HCl pH8.8, 20 mM KCl, 16 mM MgCl_2 _, 20 mM (NH_4_)_2_SO_4_, 0.1% Triton X-100), 2.6 μl of primer mixture (0.4 μM F3, 0.4 μM B3, 1.6 μM FIP, 1.6 μM BIP, 0.8 μM LPF, 0.8 μM LPB), 3.5 μl dNTPs (10 mM), 1 μl of template DNA(extracted from blood samples of rabbits) and 4.4 μl of ddH_2_O were mixed and heated at 99°C for 10 min to denature genomic DNA. After cooling on ice, 1 μl of Bst DNA polymerase (8 U; New England BioLabs) was added to this pre-mixture, incubated at 65°C for 1 h and the reaction terminated by heating to 80°C for 5 min. Successful amplification of the target gene was confirmed by 2% agarose gel electrophoresis and stained with ethidium bromide.

PCR assays were carried out in a 50 μl reaction volume containing 5 μl of 10 × reaction buffer, 5 μl MgCl_2 _(25 mM), 4 μl dNTPs (2.5 mM), 1 μl F3 (50 mM), 1 μl B3 (50 mM), 1 μl template DNA, 1 μl Taq polymerase and 32 μl ddH_2_O. Initial denaturation was carried out at 99°C for 10 min, followed by 35 cycles of denaturation (30 s at 94°C), annealing (20 s at 58°C), and extension (30 s at 72°C), with a final extension incubation for 8 min at 72°C. PCR products were visualised by 2% agarose gel electrophoresis and stained with ethidium bromide.

## Competing interests

The authors declare that they have no competing interests.

## Authors' contributions

MJ, CY and GW designed and supervised the study and critically revised the manuscript. WC and CL did the laboratory work, analyzed the data, and drafted the manuscript. Additionally, YX and HW contributed to animal infection and serum collection. HM did the part of laboratory work. All authors read and approved the final manuscript.
